# Can Ultrasound Elastography Discriminate between Rectal Adenoma and Cancer? A Systematic Review

**DOI:** 10.3390/cancers13164158

**Published:** 2021-08-18

**Authors:** Martina Kastrup Loft, Malene Roland Vils Pedersen, Hans Bjarke Rahr, Søren Rafael Rafaelsen

**Affiliations:** 1 Department of Radiology, Vejle Hospital, University Hospital of Southern Denmark, Beriderbakken 4, 7100 Vejle, Denmark; malene.roland.vils.pedersen@rsyd.dk (M.R.V.P.); Soeren.Rafael.Rafaelsen@rsyd.dk (S.R.R.); 2 Department of Regional Health Research, University of Southern Denmark, Campusvej 55, 5000 Odense, Denmark; hans.rahr@rsyd.dk; 3 Danish Colorectal Cancer Center South, Vejle Hospital, University Hospital of Southern Denmark, 7100 Vejle, Denmark; 4 Department of Surgery, Vejle Hospital, University Hospital of Southern Denmark, Beriderbakken 4, 7100 Vejle, Denmark

**Keywords:** rectal neoplasms, elastography, ultrasound

## Abstract

**Simple Summary:**

It is important to obtain correct preoperative classification of rectal polyps and cancer prior to making treatment decisions but distinguishing between the two can be challenging. International societies recommend magnetic resonance imaging and endorectal ultrasound as part of the initial preoperative evaluation. The ultrasound examination can be improved by applying a tissue stiffness measurement, known as elastography. Our aim was to investigate the performance of elastography in the staging of rectal tumors. We systematically searched the literature and found six eligible studies. They all reported increased accuracy by elastography compared with ultrasound examination alone.

**Abstract:**

Background: Rectal cancer is a common malignancy. Since the introduction of bowel-screening programs, the number of patients with advanced adenomas and early rectal cancer has increased. Despite improved diagnostics, the discrimination between rectal adenomas and early rectal cancer (i.e., pT1–T2) remains challenging. The purpose of this systematic review was to evaluate the diagnostic performance of endorectal ultrasound (ERUS) elastography in discriminating rectal adenomas from cancer. Method: Using PRISMA guidelines, a systematic search was performed on PubMed, Embase, and MEDLINE databases. Studies evaluating the primary staging of rectal adenomas and cancer using ERUS elastography were included. Results: Six studies were identified; three evaluated the discrimination between adenomas and cancer; two evaluated adenomas and early rectal cancer (i.e., pT1–T2); one evaluated performance on different T categories. All studies reported increased diagnostic accuracy of ERUS elastography compared to ERUS. Sensitivity, specificity and accuracy ranged 0.93–1.00, 0.83–1.00 and 0.91–1.00, respectively, when discriminating adenomas from cancer. In the differentiation between adenomas and early rectal cancer, the sensitivity, specificity and accuracy were 0.82–1.00, 0.86–1.00 and 0.84–1.00, respectively. Conclusion: Elastography increases the accuracy of ERUS and may provide valuable information on malignant transformation of rectal lesions.

## 1. Introduction

The introduction of screening programs for bowel cancer in many countries has increased the number of patients diagnosed with early rectal cancers [1,2,3] and advanced adenomas [4]. The treatment of rectal tumors spans a wide variety of modalities, from simple endoscopic removal to combinations of extensive oncological treatment and major surgery. The current goals are individualized treatment and organ preservation, i.e., the avoidance of major surgery except when absolutely necessary. This calls for better diagnostic methods to allocate the patient to the adequate treatment. Various approaches may be taken to this problem. Improvements in endoscopic technology have shown promising results in expert hands [5], but is still not used routinely. Another approach would be the improvement of radiologic imaging techniques. Currently, magnetic resonance imaging (MRI) is the first choice of technique [6] when suspecting rectal malignancy, especially when categorizing advanced tumors and assessing invasion of the tumor into the pelvic structures [7]. However, MRI tends to overstage cT1-2 tumors, potentially resulting in missed opportunities for organ preservation treatment [8]. Therefore, an additional endorectal ultrasonography (ERUS) is recommended when categorizing early rectal cancer [9,10]. Although ERUS is considered to have a high accuracy in rectal cancer [11], others suggest that the accuracy of ERUS in distinguishing benign adenomas from T1 rectal cancer is limited [12]. In significant colorectal polyps >2 cm, malignant transformation has been reported in approximately 20% [13,14] resulting in an increased risk of local recurrence [15].

A recent addition to increase the accuracy of ERUS is ultrasound elastography (UE). By assessing the tissue stiffness within the lesion, this technique provides information on tissue elasticity, which corresponds to the malignant transformation [16] and may aid in the categorization and discrimination between rectal adenoma and cancer.

The objective of this study was to determine the diagnostic accuracy of ERUS elastography in distinguishing rectal adenomas from rectal cancer.

Furthermore, we aimed to investigate its diagnostic accuracy in differentiating adenomas from early rectal cancer.

## 2. Methods

The Preferred Reporting Items for Systematic Review and Meta-Analysis (PRISMA) [17] guided this systematic review and the reporting of its findings.

### 2.1. Ultrasound Elastography

**Figure 1:**
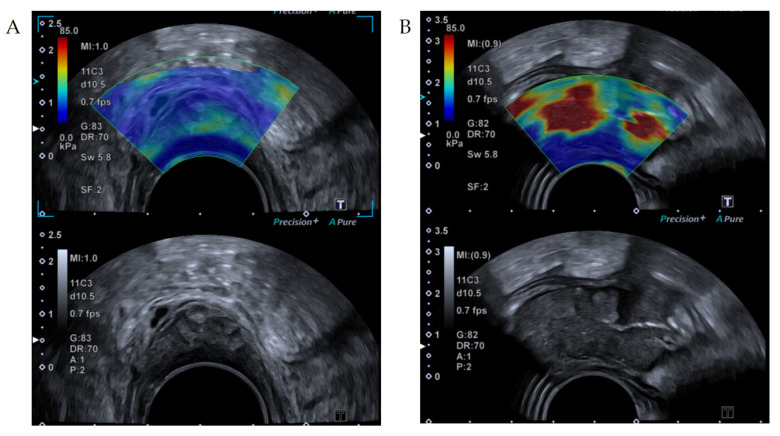
Shear wave elastography images of an (**A**) adenoma and a (**B**) adenocarcinoma. Upper image shows the elastogram superimposed with a B-mode image. Bottom image is the corresponding B-mode ultrasound image allowing the examiner to place the region of interest within the tumor area. (**A**): The elastogram shows soft values (blue and green colors). (**B**): Adenocarcinoma with high elastography values (red colors).

**Figure 2:**
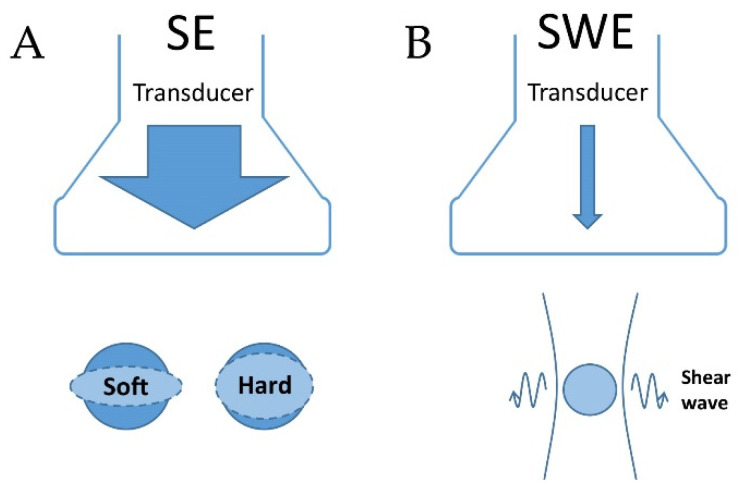
Methods for strain elastography and shear wave elastography. (**A**): Principle of strain elastography (SE) using manual compression. A soft tumor compresses more than a hard tumor. (**B**): principle of shear wave elastography (SWE) using acoustic radiation impulses created by the transducer. Shear waves are detected as they pass through the tissue.

Elastography techniques measure tissue elasticity. When tissue undergoes a type of deformation (change of shape) known as shear due to applied force, the elastic restoring forces of the tissue will act against the deformation. Shear deformation is observed using ultrasound imaging and converted into either an elasticity image (elastogram) or a local measurement (elastography value). Thus, the principle of elastography is to apply a force and measure and map the generated tissue displacement. The elastogram may be separately displayed or superimposed with a B-mode scan distinguished using colors (Figure 1). The generated displacement of tissue is directly dependent on the value of shear modulus G, for a given force, and hence converted into either strain (calculated from the percentage deformation) or time of arrival from the displacement profiles i.e., displacement over time to derive shear wave speed [18]. Multiple methods of UE are commercially available, but when it comes to ERUS elastography, only strain elastography (SE) and shear wave elastography (SWE) have been used in published articles. The methods of SE and SWE are shown in Table 1 and visualized in Figure 2.

#### 2.1.1. Strain Elastography

SE is a qualitative technique. To create SE imaging, a mechanical force is applied, using the ultrasound transducer or a water-filled balloon to displace the tissue. This is referred to as quasi-static compression. The mechanical force should be applied with a slow palpation rate relative to the propagation time to the depth. The generated tissue displacement is converted into strain calculated from the percentage of deformation. Although the absolute stiffness of the lesion is unknown, the ratio of the relative stiffness to normal tissue stiffness, known as the strain ratio (SR), is used for lesion characterization.

#### 2.1.2. Shear Wave Elastography

SWE is a quantitative technique with the deformation force generated by the ultrasound probe as acoustic radiation impulses. The subsequent monitoring of the displacement of tissue elements is a function of time as a shear wave passes multiple points along its path. Hence, SWE is dependent on shear wave speed through the tissue and is measured in either meters per second or due to the close relationship between shear modulus G and Young’s modulus E in kilo Pascal (kPa). With SWE the absolute stiffness of the tissue is obtained and can be used to characterize the lesion as can the ratio between lesion and normal tissue.

### 2.2. Search Strategy

A university librarian assisted systematic literature search was performed on the PubMed, Embase and MEDLINE (Ovid) databases on 7 January 2021. The search included keywords corresponding to the target condition, rectal neoplasms (or equal term), in combination with elasticity imaging techniques or elastography. A detailed search query is presented in Table S1. No restrictions were applied during the literature search. Relevant studies on the diagnostic performance of ERUS elastography in the staging of primary rectal cancer were thus identified. The reference lists of all identified articles eligible for full text evaluation were checked for additional relevant publications.

### 2.3. Study Selection

Two reviewers (M.K.L. and M.R.V.P.) searched the databases independently and checked the eligibility of each identified article based on titles and/or abstracts. Eligible studies were finally included after full-text evaluation performed independently by the two reviewers. Any disagreement was resolved by consensus through discussion with a third reviewer (S.R.R.).

Publications were eligible if they assessed the diagnostic performance of ERUS elastography in the differentiation between rectal adenomas and cancer.

The inclusion criteria were as follows: (1) Adult patients (age ≥ 18) with a rectal lesion; (2) studies performing elastography using the ERUS approach; (3) purpose of study to assess the diagnostic accuracy of ERUS elastography in distinguishing between benign and malignant rectal lesions; (4) biopsy or surgical specimen serving as end-point.

Exclusion criteria were (1) non-original research articles, including reviews, case reports, editorials, commentaries, letters and conference abstracts; (2) recurrent rectal cancer; (3) non-English publications.

### 2.4. Data Extraction and Quality Assessment

Two reviewers (M.K.L. and M.R.V.P.) performed quality assessment and data extraction independently. Any disagreement was resolved by reaching consensus. If consensus could not be reached, a third author (S.R.R.) was consulted. The following data were extracted from the selected studies: (1) Study characteristics: First author, year of publication, duration of patient recruitment, study design, consecutive enrollment; (2) patient characteristics: Sample size, age, gender; (3) elastography characteristics: Number of readers, operator experience in ERUS elastography, ultrasonography machine, type of probe, elastography technique, mode of measurement, mean diameter of region of interest (ROI), number of measurements; (4) histopathological characteristics: Biopsy or surgical specimen, time interval between elastography assessment and surgery; (5) details on diagnostic performance (e.g., sensitivity and specificity).

Forest plots present the point estimates of sensitivity and specificity in each study.

Assessment of the methodological quality of each study was performed according to the Quality Assessment of Diagnostic Accuracy Studies-2 (QUADAS-2) [19] tool. Questions and background information of the QUADAS-2 tool is available at the QUADAS web site (www.quadas.org, accessed on 10 February 2021). Each study was assessed as having a ‘low’ (all question answered yes), ‘high’ (at least one answered no) or ‘unclear’ (one answer unclear) risk of bias within the following four domains: Patient characteristics; index test; reference standard; flow and timing. Concerns regarding applicability were assessed in three domains: Patient characteristics and setting; index test; reference standard. In relation to risk of bias assessment, a question was added to clarify whether the index test was performed blinded to patient symptoms and endoscopy findings. 

The methodological quality of the studies was assessed as reported. We did not contact authors to clarify methodological uncertainties, and therefore the quality may not always fully reflect the study as was conducted.

A meta-analysis was not performed due to study heterogeneity and a small sample size.

## 3. Results

### 3.1. Literature Search

**Figure 3:**
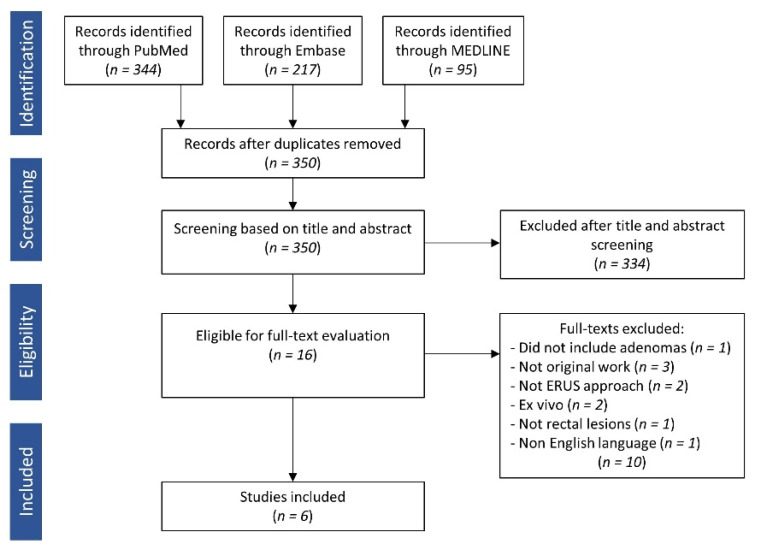
PRISMA flowchart describing the identification and inclusion of studies.

The initial search yielded 656 studies. The removal of duplicates left 350 publications of which 334 were excluded after title and abstract screening. The remaining 16 studies were evaluated in full text. Ten were excluded due to adenomas not included (1); non-original data (3); trans-gluteal approach (2); ex vivo design (2); no rectal lesions (1); Chinese language (1). Two papers investigated the same study population but addressed different issues. Since they matched either of our review questions, they were both included. A total of six studies were finally included. A flow-chart is presented in Figure 3.

### 3.2. Quality of Included Studies

**Figure 4:**
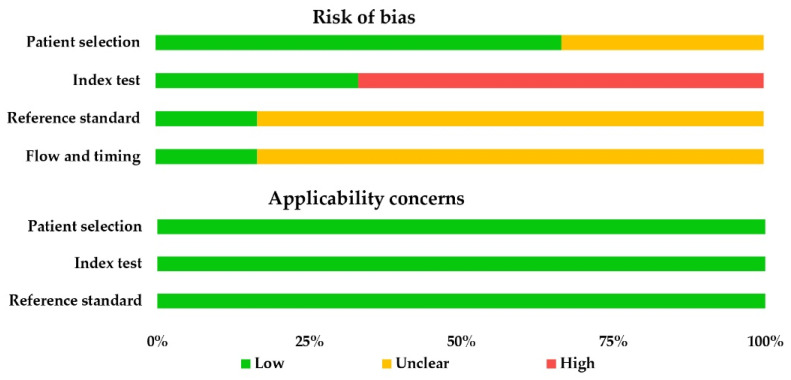
QUADAS-2 score of all studies combined.

The QUADAS-2 checklist of the six included studies is presented in Table 2. There were no severe concerns regarding applicability. Only one study reported a time interval between the index test and reference standard. All studies evaluating SE were conducted with the knowledge of patient symptoms and endoscopy findings. The QUADAS-2 score of all studies combined is presented in Figure 4.

### 3.3. Study Characteristics

All studies were single center with a prospective design and consecutive enrollment. The populations varied from 37 to 115 patients. Four studies used SE of which three [23,24,25] defined an SR cut-off value for malignancy of ≥1.25 and one [20] used SR < 0.8 as benign and ≥1.6 as malignant. In the latter study, patients with SR between 0.8 and 1.6 were considered inconclusive and hence were excluded. Two studies evaluated SWE. One [22] used a pilot cohort to define a cut-off value for malignancy of ≥26.9 kPa and subsequently evaluated a validation cohort. The other study [21] defined lesions by color with predominantly red/yellow lesions considered to be an indication of malignancy and blue/green as benign.

Three of six studies [20,22,23] evaluated the differentiation between benign and early malignant tumors (pT1 and pT2), two of which [20,23] focused on benign and early rectal cancer and one [22] on differentiation of T categories, including adenomas (pT0).

Individual study characteristics of the population and equipment characteristics related to the elastography procedure are shown in Table 3 and Table 4, respectively.

### 3.4. Diagnostic Accuracy and Staging

All six studies reported an increase in diagnostic accuracy by elastography compared with ERUS (Table 5).

In four of six studies [21,22,24,25], the sensitivity and specificity of UE in differentiating benign from malignant lesions was reported or calculated from 2 × 2 contingency tables. Two of them evaluated SE and two SWE. The SE studies used comparable methods and thresholds. The studies performing SWE used different thresholds as described previously. The total study population was 379 patients; 183 were evaluated performing SE and 196 using SWE.

The point estimates of sensitivity and specificity in each study are shown in Table 6. As also shown, the range of sensitivity and specificity was 0.93–1.00 and 0.83–1.00, respectively.

### 3.5. Accuracy of ERUS Elastography in the Differentiation of Adenomas and Early Rectal Cancer

Two of six studies [20,23] evaluated the performance of elastography in differentiating adenomas from early rectal cancer (pT1 and pT2). The study by Chen et al. was also included, as the sensitivity and specificity regarding early rectal cancer was calculated from 2 × 2 contingency tables. The total study population was 152 patients; 115 from two studies were evaluated using SE and 37 using SWE. One SE study [23] defined an SR cut-off of ≥1.25, the other [20] cut-off values of <0.8 for benign and ≥1.6 for malignant lesions. Thus, the two SE studies used similar techniques with different thresholds. The study evaluating SWE defined a cut-off value of ≥26.9 kPa.

The point estimates of sensitivity and specificity in each study are shown in Table 7 with sensitivity and specificity ranging 0.82–1.00 and 0.86–1.00, respectively.

## 4. Discussion

This systematic review shows that ERUS elastography increases the diagnostic accuracy of discriminating rectal adenomas from malignant tumors compared with ERUS, as seen in Table 5. The increased accuracy was independent of elastography technique.

We found only a limited number of studies in the literature, however, addressing the aspect of discriminating benign from early rectal malignancies. The studies unanimously showed an increase in accuracy when compared with ERUS, although with lower sensitivity, specificity, PPV, NPV and accuracy compared to studies including both early and advanced tumor stages. A possible explanation is that the stiffness of malignant rectal tissue increases with T category [22,26]. Hence, advanced malignant tumors yield higher elastography values than adenomas and early carcinomas. Another aspect is size, since a large, advanced tumor contains an increased amount of stiffer tissue [27]. In general, this may explain why studies including T3 and T4 tumors report higher accuracy. For that reason, patient selection becomes an important issue when assessing the usefulness of the studies. From a clinical point of view, the ability of an investigation to distinguish between benign lesions and early cancers will have a much greater impact on treatment than its ability to confirm that an advanced and obviously malignant tumor is not an adenoma.

UE has been shown to provide valuable information on the malignant transformation of tissue in other organs such as the breast, thyroid and prostate [16,28]. Preoperative evaluation of advanced adenomas and cancer is imperative in the treatment strategy planning. Multiple treatment options are available ranging from minimally invasive surgical procedures to major resection and chemoradiotherapy [29]. Treatment options are discussed at multidisciplinary team (MDT) meetings with dedicated specialists representing surgery, oncology, pathology and radiology [30]. MRI is the first choice for radiological staging of a primary rectal tumor. The modality yields a high accuracy in advanced tumors (T3-T4) [6], but is reported to overstage early rectal malignancies [8], thus potentially resulting in missed opportunity for organ preservation treatment, e.g., transanal endoscopic microsurgery (TEM) or the watchful waiting strategy (WW). ERUS is considered superior to MRI in the staging of T1 and T2 rectal malignancies, and, consequently, the international guidelines recommend supplemental ERUS when evaluating advanced adenomas or early rectal malignancies [9].

Regarding the applicability of ERUS elastography in routine practice, it should first and foremost be noted that the ERUS procedure itself is operator dependent [31,32] and a minimum experience of 50 procedures is recommended [33]. UE is an add-on to the ERUS and, consequently, also operator dependent. Comparisons of SE and SWE have been done for breast lesions, but without finding significant differences [34]. Endoluminal examinations, however, may be a different situation. SWE is recommended for prostate gland evaluation owing to a shorter learning curve and less intra- and inter-operator variation, but no studies compared SE and SWE head-to-head [28,35]. The same recommendation may not apply to evaluating rectal tumors. Both intra- and inter-operator agreement of SE and SWE are reported to be high in rectal tumors, but direct comparisons have not been reported [21,36]. In contrast to lesions in the prostate, rectal tumors appear close to the transducer, avoiding depth artifacts that can occur in a large prostate. With the limited literature available, the superiority of one method above the other cannot be established; however, the results appear to be quite similar. We found that adding elastography (any method) to ERUS increased the accuracy, and we would recommend to add UE to improve the accuracy of ERUS, provided that the operators are familiar with the limitations of the specific UE method [18]. It is beyond the scope of the current review to compare ERUS elastography with other clinical strategies, but its accuracy seems to compare well with advanced endoscopy technologies like magnification endoscopy [5,37]. Which strategy to choose may depend on local expertise and logistics. Finally, treatment decisions are always based on an overall assessment of all available information, including imaging results and endoscopic appearance and histopathology of the tumor itself, as well as clinical information on the patient’s condition and preferences.

The two SWE studies [21,22] included in this review used the Aixplorer (SuperSonic Imaging), but the predefined thresholds were incomparable. Li et al. used a visual approach with predominantly red and yellow colors considered predictors of malignancy. Hence, an evaluation of the SWE stiffness value was calculated yielding a mean and max cut-off value of 61.3 kPa and 63.4 kPa, respectively. The corresponding sensitivity and specificity were 0.89–0.94 and 0.88–0.83, respectively. Chen et al. defined a mean cut-off value of 26.9 kPa based on a pilot cohort of 70 patients, 11 of which were diagnosed with adenoma. In the subsequent validation cohort of 30 patients, only 3 had adenoma. When discriminating benign tissue from early rectal malignancy, only a limited number of patients from the study were eligible for inclusion. This could explain why Chen et al. achieved sensitivity, specificity and accuracy of 100%.

All SE studies used equipment from the same manufacturer and the same approach. The operator used a water-filled balloon covering the transducer connected to a syringe to induce tissue compression decompression. It may be a challenge, however, to secure the same inter-operator compliance in relation to amount of pressure and frequency. Variation between different segments of the rectum should also be taken into account.

The two SE studies [20,23] addressing benign versus early rectal malignancy used different thresholds. Waage et al. (2015a) used an SR ≥ 1.25 as prediction of malignancy and subsequently proposed the thresholds of SR < 0.8 for benign tissue, SR ≥ 1.60 for malignant and SR 0.8–1.60 for inconclusive lesions. Oien et al., using the thresholds proposed by Waage et al. (2015a), observed no difference in sensitivity and specificity, and Oien et al. reported lower values of PPV (0.88 versus 0.97) and accuracy (0.84 versus 0.94). A possible explanation could be the setting and number of patients and readers. Whereas the study by Waage et al. (2015a) was conducted in a scientific setting with only one reader, Oien et al. focused on the elastography procedure in a clinical setting with multiple readers of varying experience. In a clinical setting, the operators are not blinded to previous examinations or biopsy results, thereby reflecting the everyday clinic.

## 5. Strengths and Limitations

In this PRISMA review, a meta-analysis was not performed due to a limited number of eligible studies and the heterogeneity of the elastography methods.

Different cut-off values were proposed in both SE and SWE. For SE, an SR cut-off value of ≥1.25 was initially proposed and later adjusted to <0.8 as benign and ≥1.6 as malignant. The two SWE studies propose mean values of 26.9 kPa and 61.3 kPa, respectively. A uniform SWE cut-off value could not be extracted due to the limited number of studies and included patients, and none of the SWE studies were performed with the objective to discriminate benign tissue from early rectal malignancy. Furthermore, different ultrasound equipment manufacturers use different elastogram algorithms, and cut-off values must be validated before being applied in clinical practice. Only the study by Oien et al. evaluated the use of elastography in a clinical setting. Studies reporting treatment decisions are few. Future large, well-designed studies addressing this area should report clinical endpoints, e.g., impact on treatment decisions, and should evaluate and compare SE and SWE. This may expand the potential of EU to become an important imaging modality in rectal tumors. The results of this review indicate that morphological changes in a tumor increase tissue stiffness, suggesting the principle of elastography algorithms to be applicable in the staging of rectal tumors.

## 6. Conclusions

Elastography shows potential for discrimination between rectal adenoma and cancer. All studies report increased tissue stiffness measured with elastography in malignant tumors. Notably, few studies address the ability to discriminate between adenoma and early rectal cancer, and large-scale studies on the subject are warranted.

## Figures and Tables

**Table 1:** Methods for strain elastography and shear wave elastography.

Method	Type of Force	Applied Force	Property Displacement	Qualitative/Quantitative	Imaging/Measurement
SE	Quasi static	Mechanically induced	Strain rate	Qualitative	Full area
SWE	Dynamic	Acoustic radiation force	Shear wave speed	Quantitative	Image within a color box

SE, Strain elastography; SWE, Shear wave elastography.

**Table 2:** QUADAS-2 Checklist for all included studies.

Study	Risk of Bias				Applicability Concerns		
	Patient Selection	Index Test	Reference Standard	Flow and Timing	Patient Selection	Index Test	Reference Standard
Oien_2019 [20]	Low	High	Unclear	Unclear	Low	Low	Low
Li_2019 [21]	Unclear	Low	Unclear	Unclear	Low	Low	Low
Chen_2017 [22]	Unclear	Low	Low	Low	Low	Low	Low
Waage_2015a [23]	Low	High	Unclear	Unclear	Low	Low	Low
Waage_2015b [24]	Low	High	Unclear	Unclear	Low	Low	Low
Waage_2011 [25]	Low	High	Unclear	Unclear	Low	Low	Low

**Table 3:** Study characteristics of the included articles.

Author_Year	Duration of Patient Recruitment	Sample Size *n*:	Benign *n*_b_:	Malignant (pT1-T2) *n*_m_:	Age, Years Median (Range) Mean Age ± SD	Sex	Reference Standard	Elastography -Reference Time Interval	Research Question
Oien_2019 [20]	10/2014–1/2018	96	45	(51)	median: 68 (31–91)	M/F ratio: 1,64	Surgical specimen	NR	Benign vs. pT1-2
Li_2019 [21]	12/2016–2/2018	96	24	72	mal: 60.8 ± 9.7 ben: 55.9 ± 13.7	M/F: 55/41	Surgical specimen or biopsy	NR	Benign vs. malignant
Chen_2017 [22]	4/2015–7/2016	100	14	86 (23)	pilot: 60 ± 12 val.: 59 ± 11	M/F: 60/40	Surgical specimen	≤2 weeks	Cut-off values T-stages
Waage_2015a [23]	11/2009–4/2011	43	21	(22)	mean: 69 *	NR	Surgical specimen	NR	Benign vs. pT1-2
Waage_2015b [24]	11/2009–4/2011	115	21	94	median: 66 (25–88)	M/F: 67/53 *	Surgical specimen or biopsy	NR	Benign vs. malignant
Waage_2011 [25]	4/2008–9/2009	68	23	45	median: 70 (35–92)	M/F: 42/26	Surgical specimen	NR	Benign vs. malignant

*n*_b_, number at pathologically benign lesions; *n*_m_, number of pathologically malignant lesions; NR, Not reported; M/F, Male/Female; mal, population with a malignant lesion; ben, population with a benign lesion; SD, standard diviation; vs., versus; pilot, mean age ± SD of the population in the pilot cohort; val, mean age ± SD of population in the validation cohort; * Age/sex is based on the entire population of the study, not the subpopulation we extracted.

**Table 4:** Ultrasound elastography characteristics of included studies.

Author _Year	Number of Readers	Operator Experience	Blinding of Endoscopy and/or Image Findings	US Machine	Probe (MHz)	ELASTOGRAPHY Technique	Diameter of ROI	Number of Measurements	Method of Processing Measurements
Oien_2019 [20]	7	Variable	No	Hitachi EUB-8500	360° rigid probe EUP-R54AW-19 (5–10 MHz)	SE	NR	5	Median
Li_2019 [21]	2	Experienced examiner	Yes	Aixplorer (SuperSonic Imagine)	Endfire probe SE12-3 (8 MHz)	SWE	3–10 mm	1	Color *
Chen_2017 [22]	1	At least 100 ERUS	Yes	Aixplorer (SuperSonic Imagine)	Endfire probe SE12-3 (8 MHz)	SWE	NR	5	Mean
Waage_2015a [23]	1	NR	No	Hitachi EUB-8500	360° rigid probe EUP-R54AW-19 (10 MHz)	SE	NR	NR	Mean
Waage_2015b [24]	1	NR	No	Hitachi EUB-8500	360° rigid probe EUP-R54AW-19 (10 MHz)	SE	NR	5	Mean
Waage_2011 [25]	1	NR	No	Hitachi EUB-8500	360° rigid probe EUP-R54AW-19 (5–10 MHz)	SE	NR	3	Mean

US, ultrasound; UE, ultrasound elastography; ROI, region of interest; SE, strain elastography; SWE, shear wave elastography; NR, not reported; * Color: Lesions were assessed based on color, predominantly red and yellow colors were considered malignant, predominantly green and blue colors were considered benign.

**Table 5:** Accuracy of ultrasound elastography versus other reported modalities for diagnosis of rectal cancer.

Study	Tumor Categorization ¤ Benign; Early; Advanced ^#^	Sensitivity (95% CI)	Specificity (95% CI)	PPV	NPV	Accuracy
Oien_2019 [20]						
ERUS + UE (*n* = 96)	47%; 53%; -	0.82 (0.69–0.91)	0.87 (0.73–0.94)	0.88	0.81	0.84
ERUS (*n* = 127)		0.70	0.92	0.90	0.74	0.80
MRI (*n* = 84)		0.98	0.16	0.73	0.80	0.74
Li_2019 [21]						
ERUS + UE (*n* = 96)	25%; 49%; 26%	0.93 (0.85–0.98)	0.83 (0.63–0.95)	0.94 (0.86–0.98)	0.80 (0.59–0.93)	0.91
ERUS (*n* = 96)		0.89 (0.77–0.95)	0.79 (0.58–0.93)	0.93 (0.84–0.98)	0.70 (0.50–0.86)	0.86
Chen_2017 [22]						
UE (*n* = 100) *	14%; 23%; 63%	1.00 (0.95–1.00) ^#^	1.00 (0.73–1.00) ^#^	1.00 (0.95–1.00) ^#^	1.00 (0.73–1.00) ^#^	1.00 ^#^
ERUS (*n* = 100) *		0.97 (0.89–0.99) ^#^	0.71 (0.42–0.90) ^#^	0.95 (0.88–0.99) ^#^	0.77 (0.46–0.94) ^#^	0.93 ^#^
Waage_2015a † [23]						
UE (*n* = 42)	49%; 51%; -	0.82 (0.61–0.94)	0.86 (0.66–0.96)	0.86 (0.66–0.96)	0.82 (0.61–0.94)	0.84 (0.71–0.93)
ERUS (*n* = 42)		0.82 (0.61–0.94)	0.62 (0.40–0.80)	0.69 (0.49–0.85)	0.76 (0.51–0.92)	0.72 (0.58–0.85)
Waage_2015b † [24]						
UE (*n* = 115)	18%; 19%; 63%	0.96 (0.90–0.99)	0.86 (0.66–0.96)	0.97 (0.91–0.99)	0.82 (0.61–0.94)	0.94 (0.88–0.97)
ERUS (*n* = 115)		0.96 (0.90–0.99)	0.62 (0.40–0.80)	0.92 (0.85–0.96)	0.76 (0.51–0.92)	0.90 (0.83–0.94)
MRI (*n* = 108)		0.99 (0.94–1.00)	0.07 (0.00–0.31)	0.88 (0.80–0.93)	0.50 (0.03–0.97)	0.87 (0.80–0.93)
Waage_2011 [25]						
UE (*n* = 68)	34%; NR; NR	0.93 (0.81–0.98)	0.96 (0.80–1.00)	NR	NR	0.94 (0.85–0.98)
ERUS (*n* = 68)		0.91 (0.80–0.97)	0.87 (0.68–0.96)	NR	NR	0.90 (0.80–0.95)

†, duplicate cases; UE, ultrasound elastography; ERUS, endorectal ultrasound; MRI, magnetic resonance imaging; *n*, size of populations; ¤, based on data from UE; Early, pT1-T2; ^#^, pT3-T4 tumors and included patients without pathological classification e.g., due to neoadjuvant treatment; CI, confidence interval; PPV, positive predictive value; NPV, negative predictive value; NR, not reported; ^#^ Calculated from 2 × 2 contingency tables; * Based on the entire population (combined pilot and validation cohort).

**Table 6:** Ultrasound elastography discrimination between rectal adenoma and cancer, including forest plot showing sensitivity and specificity of each study.

Study	Elastography Technique	*n*:	Threshold	Sensitivity (95% CI)	Sensitivity (FP)	Specificity (95% CI)	Specificity (FP)
Li_2019 [21]	SWE	96	Color *	0.93 (0.85–0.98)	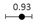	0.83 (0.59–0.93)	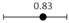
Chen_2017 [22]	SWE	100	≥26.9 kPa	1.00 (0.95–1.00) ^#^	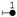	1.00 (0.73–1.00) ^#^	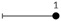
Waage_2015b [24]	SE	115	SR ≥ 1.25	0.96 (0.90–0.99)	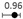	0.86 (0.66–0.96)	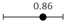
Waage_2011 [25]	SE	68	SR ≥ 1.25	0.93 (0.81–0.98)	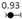	0.96 (0.80–1.00)	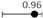

SE, strain elastography; SWE, shear wave elastography; *n*, size of populations; SR, strain ratio; CI, confidence interval; FP, Forest plot; ^#^ Calculated from 2 × 2 contingency tables; * Color: predominantly red/yellow colored lesions were considered malignant, predominantly blue/green colors were considered benign.

**Table 7:** Correlation between elastography and staging of early rectal cancer (pT1–T2).

Study	Elastography Technique	*n*:	Threshold	Sensitivity (95% CI)	Sensitivity (FP)	Specificity (95% CI)	Specificity (FP)
Oien_2019 [20]	SE	96	SR < 0.8/ ≥1.6	0.82 (0.69–0.91)	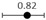	0.87 (0.73–0.94)	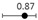
Chen_2017 [22]	SWE	37	≥26.9 kPa	1.00 (0.82–1.00) ^#^	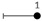	1.00 (0.73–1.00) ^#^	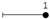
Waage_2015a [23]	SE	42	SR ≥ 1.25	0.82 (0.61–0.94)	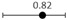	0.86 (0.66–0.96)	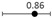

SE, strain elastography; SWE, shear wave elastography; *n*, size of population; SR, strain ratio; CI, confidence interval; FP, Forest plot; ^#^ Calculated from 2 × 2 contingency tables.

## Supplementary Materials

The following are available online at https://www.mdpi.com/article/10.3390/cancers13164158/s1, Table S1: Search query used for PubMed, Embase and MEDLINE databases.
