# Supplementary material for: Can Ultrasound Elastography Discriminate between Rectal Adenoma and Cancer? A Systematic Review
Source: Cancers (Basel). 2021 Aug 18;13(16):4158. doi: 10.3390/cancers13164158 (PMC8391413; doi:10.3390/cancers13164158)
Supplement: Supplementary file 1 [file cancers-13-04158-s001.zip › cancers-1287487-supplementary.pdf]

cancers

MDPI

Supplement to:

Can ultrasound elastography discriminate between rectal adenoma and cancer? A systematic review

Martina Kastrup Loft [1,3,4,*] , Malene Roland Vils Pedersen [1,3,4], Hans Bjarke Rahr [2,3,4] and Søren Rafael Rafaelsen [1,3,4]

[1] Department of Radiology, Vejle Hospital, University Hospital of Southern Denmark, Beriderbakken 4, Vejle, Denmark
[2] Department of Surgery, Vejle Hospital, University Hospital of Southern Denmark, Beriderbakken 4, Vejle, Denmark
[3] Department of Regional Health Research, University of Southern Denmark, Campusvej 55, Odense, Denmark
[4] Danish Colorectal Cancer Center South, Vejle Hospital, University Hospital of Southern Denmark, Demark
* Correspondence: martina.kastrup.loft@rsyd.dk

Supplement Materials: Table S1: Search query used for PubMed, Embase and MEDLINE databases.

PubMed database:

Search	**07.01.2021**	Results
#1	Rectal Neoplasms [MeSH]	48008
#2	(("rectum"[MeSH Terms] OR "rectum"[All Fields]) OR ("rectum"[MeSH Terms] OR "rectum"[All Fields] OR "rectums"[All Fields]) OR ("administration, rectal"[MeSH Terms] OR ("administration"[All Fields] AND "rectal"[All Fields]) OR "rectal administration"[All Fields] OR "rectal"[All Fields]) OR colorectal[All Fields]) AND (("neoplasms"[MeSH Terms] OR "neoplasms"[All Fields] OR "neoplasm"[All Fields]) OR ("neoplasms"[MeSH Terms] OR "neoplasms"[All Fields]) OR (("neoplasms"[MeSH Terms] OR "neoplasms"[All Fields] OR "neoplasia"[All Fields]) AND os[All Fields] AND ("neoplasms"[MeSH Terms] OR "neoplasms"[All Fields] OR "neoplasias"[All Fields])) OR ("neoplasms"[MeSH Terms] OR "neoplasms"[All Fields] OR "cancer"[All Fields]) OR ("neoplasms"[MeSH Terms] OR "neoplasms"[All Fields] OR "cancers"[All Fields]) OR ("tumour"[All Fields] OR "neoplasms"[MeSH Terms] OR "neoplasms"[All Fields] OR "tumor"[All Fields]) OR ("tumours"[All Fields] OR "neoplasms"[MeSH Terms] OR "neoplasms"[All Fields] OR "tumors"[All Fields]) OR ("tumour"[All Fields] OR "neoplasms"[MeSH Terms] OR "neoplasms"[All Fields] OR "tumor"[All Fields]) OR ("tumours"[All Fields] OR "neoplasms"[MeSH Terms] OR "neoplasms"[All Fields] OR "tumors"[All Fields]) OR ("adenocarcinoma"[MeSH Terms] OR "adenocarcinoma"[All Fields]) OR ("adenocarcinoma"[MeSH Terms] OR "adenocarcinoma"[All Fields] OR "adenocarcinomas"[All Fields]) OR ("carcinoma"[MeSH Terms] OR "carcinoma"[All Fields]) OR ("carcinoma"[MeSH Terms] OR "carcinoma"[All Fields] OR "carcinomas"[All Fields]) OR ("carcinoid tumor"[MeSH Terms] OR ("carcinoid"[All Fields] AND "tumor"[All Fields]) OR "carcinoid tumor"[All Fields] OR "carcinoid"[All Fields]) OR malignant[All Fields] OR ("neoplasms"[MeSH Terms] OR "neoplasms"[All Fields] OR "malignancy"[All Fields]) OR ("neoplasms"[MeSH Terms] OR "neoplasms"[All Fields] OR "malignancies"[All Fields]) OR ("polyps"[MeSH Terms] OR "polyps"[All Fields] OR "polyp"[All Fields]) OR ("polyps"[MeSH Terms] OR "polyps"[All Fields]) OR ("adenoma"[MeSH Terms] OR "adenoma"[All Fields]) OR ("adenoma"[MeSH Terms] OR "adenoma"[All Fields] OR "adenomas"[All Fields]) OR benign[All Fields])	227083
#3	#1 OR #2	231245
#4	Elasticity imaging techniques [MeSH]	8774
#5	("elasticity imaging techniques"[MeSH Terms] OR ("elasticity"[All Fields] AND "imaging"[All Fields] AND "techniques"[All Fields]) OR "elasticity imaging techniques"[All Fields] OR "elastography"[All Fields]) OR ("elasticity imaging techniques"[MeSH Terms] OR ("elasticity"[All Fields] AND "imaging"[All Fields] AND "techniques"[All Fields]) OR "elasticity imaging techniques"[All Fields] OR "elastographies"[All Fields]) OR elastographic[All Fields] OR ("elasticity"[MeSH Terms] OR "elasticity"[All Fields]) OR elasticities[All Fields] OR ("elasticity imaging techniques"[MeSH Terms] OR ("elasticity"[All Fields] AND "imaging"[All Fields] AND "techniques"[All Fields]) OR "elasticity imaging techniques"[All Fields] OR "elastogram"[All Fields]) OR ("elasticity imaging techniques"[MeSH Terms] OR ("elasticity"[All Fields] AND "imaging"[All Fields] AND "techniques"[All Fields]) OR "elasticity imaging techniques"[All Fields] OR "elastograms"[All Fields]) OR sonoelastogram[All Fields] OR sonoelastograms[All Fields] OR sonoelastographic[All Fields] OR ("elasticity imaging techniques"[MeSH Terms] OR ("elasticity"[All Fields] AND "imaging"[All Fields] AND "techniques"[All Fields]) OR "elasticity imaging techniques"[All Fields] OR "sonoelastography"[All Fields]) OR ("elasticity imaging techniques"[MeSH Terms] OR ("elasticity"[All Fields] AND "imaging"[All Fields] AND "techniques"[All Fields]) OR "elasticity imaging techniques"[All Fields]) OR ARFI[All Fields] OR (("acoustics"[MeSH Terms] OR "acoustics"[All Fields] OR "acoustic"[All Fields]) AND ("radiation"[MeSH Terms] OR "radiation"[All Fields] OR "electromagnetic radiation"[MeSH Terms] OR ("electromagnetic"[All Fields] AND "radiation"[All Fields]) OR "electromagnetic radiation"[All Fields]) AND force[All Fields]) OR ("elasticity imaging techniques"[MeSH Terms] OR ("elasticity"[All Fields] AND "imaging"[All Fields] AND "techniques"[All Fields]) OR "elasticity imaging techniques"[All Fields] OR ("vibro"[All Fields] AND "acoustography"[All Fields]) OR "vibro	134782

	acoustography"[All Fields]) OR ("elasticity imaging techniques"[MeSH Terms] OR ("elasticity"[All Fields] AND "imaging"[All Fields] AND "techniques"[All Fields]) OR "elasticity imaging techniques"[All Fields]) OR acoustography[All Fields] OR acoustographic[All Fields]	
#6	#4 OR #5	134782
#7	#3 AND #6	**344**

Embase database:

Search	**07.01.2021**	Results
#1	exp rectum cancer/	231184
#2	((rect* or colorec*) adj7 (neoplas* or cancer* or carc* or tumo* or adenocarc* or polyp* or adenom* or malign* or benign*)).mp. [mp=title, abstract, heading word, drug trade name, original title, device manufacturer, drug manufacturer, device trade name, keyword, floating subheading word, candidate term word]	333188
#3	#1 or #2	333554
#4	exp elastography/	29803
#5	elasticit* or ARFI or (acouistic adj3 radiation adj3 force) or vibro-acoustograph* or (vibro adj3 acoustograph*) or elastogra* or sonoelastogr* or acoustogra*.mp. [mp=title, abstract, heading word, drug trade name, original title, device manufacturer, drug manufacturer, device trade name, keyword, floating subheading word, candidate term word]	74298
#6	#4 or #5	84210
#7	#3 and #6	**217**

MEDLINE (Ovid) database:

Search	**07.01.2021**	Results
#1	exp Rectal Neoplasms/	47985
#2	((rect* or colorec*) adj7 (neoplas* or cancer* or carc* or tumo* or adenocarc* or polyp* or adenom* or malign* or benign*)).mp. [mp=title, abstract, original title, name of substance word, subject heading word, floating sub-heading word, keyword heading word, organism supplementary concept word, protocol supplementary concept word, rare disease supplementary concept word, unique identifier, synonyms]	202374
#3	#1 or #2	207314
#4	exp Elasticity Imaging Techniques/	8768
#5	(elasticit* or ARFI or (acouistic adj3 radiation adj3 force) or vibro-acoustograph* or (vibro adj3 acoustograph*) or elastogra* or sonoelastogr* or acoustogra*).mp. [mp=title, abstract, original title, name of substance word, subject heading word, floating sub-heading word, keyword heading word, organism supplementary concept word, protocol supplementary concept word, rare disease supplementary concept word, unique identifier, synonyms]	64092
#6	#4 or #5	64092
#7	#3 and #6	**95**
